# The genetics of virus particle shape in equine influenza A virus

**DOI:** 10.1111/irv.12197

**Published:** 2013-11-14

**Authors:** Debra Elton, Emily A. Bruce, Neil Bryant, Helen M. Wise, Shona MacRae, Adam Rash, Nikki Smith, Matthew L. Turnbull, Liz Medcalf, Janet M. Daly, Paul Digard

**Affiliations:** ^1^ Animal Health Trust Suffolk UK; ^2^ Division of Virology Department of Pathology University of Cambridge Cambridge UK; ^3^ The Roslin Institute University of Edinburgh Midlothian UK; ^4^ Present address: School of Veterinary Medicine & Science University of Nottingham Sutton Bonington UK; ^5^ Present address: Department of Microbiology Perelman School of Medicine University of Pennsylvania Philadelphia PA USA

**Keywords:** Budding, filamentous, influenza, matrix

## Abstract

**Background:**

Many human strains of influenza A virus produce highly pleomorphic virus particles that at the extremes can be approximated as either spheres of around 100 nm diameter or filaments of similar cross‐section but elongated to lengths of many microns. The role filamentous virions play in the virus life cycle remains enigmatic.

**Objectives/Methods:**

Here, we set out to define the morphology and genetics of virus particle shape in equine influenza A virus, using reverse genetics and microscopy of infected cells.

**Results and Conclusions:**

The majority of H3N8 strains tested were found to produce filamentous virions, as did the prototype H7N7 A/eq/Prague/56 strain. The exception was the prototype H3N8 isolate, A/eq/Miami/63. Reassortment of equine influenza virus M genes from filamentous and non‐filamentous strains into the non‐filamentous human virus A/PR/8/34 confirmed that segment 7 is a major determinant of particle shape. Sequence analysis identified three M1 amino acid polymorphisms plausibly associated with determining virion morphology, and the introduction of these changes into viruses confirmed the importance of two: S85N and N231D. However, while either change alone affected filament production, the greatest effect was seen when the polymorphisms were introduced in conjunction. Thus, influenza A viruses from equine hosts also produce filamentous virions, and the major genetic determinants are set by the M1 protein. However, the precise sequence determinants are different to those previously identified in human or porcine viruses.

## Introduction

Influenza A virus (IAV) is a major pathogen of homoeothermic vertebrates, affecting man and a variety of wild and domesticated mammalian and avian species, with aquatic birds probably representing the reservoir host.^1^ The virus forms enveloped virions containing nine structural proteins and the eight segments of negative‐sense single‐stranded RNA that comprise the genome by budding through the apical plasma membrane.^2^ Three viral transmembrane proteins are included in the envelope (haemagglutinin (HA), neuraminidase and M2 ion channel), linked via their cytoplasmic domains to an internal layer of a matrix (M1) protein which in turn interacts with the genomic ribonucleoproteins.

Early studies on the morphology of human strains of virus established that two types of particle were formed: pleomorphic spheres of approximately 100 nm diameter or elongated forms of the same diameter, but reaching many microns in length.^3^, ^4^ Both forms of virus particle are infectious, and although there are preparation‐dependent variations in particle:infectivity ratios, the overall values for filaments and spheres are similar.^5^, ^6^ The majority of low‐passage human IAV strains are filamentous in cell culture, although this is a trait that can be lost upon prolonged passage in the laboratory.^3^, ^5^, ^7^ More recent work has established that the morphology of virus particles is determined by both viral and cellular factors. With respect to the virus, segment 7 in particular, sequence polymorphisms in the M1 gene play a dominant role.^7^, ^8^, ^9^, ^10^ From the cellular side, epithelial cells tend to produce more and/or larger filaments than fibroblasts, and an intact actin cytoskeleton is required for filament but not for spherical particle formation.^11^, ^12^ The endocytic trafficking regulator Rab11 and its effector protein Rab11‐FIP3 are also required for filamentous budding.^13^ However, the biological significance of the filamentous forms remains unclear. Although formation of short filaments in infected human lung explants has recently been reported,^14^ convincing demonstration that filaments are formed *in vivo* is lacking, and their significance in pathogenicity in animal models has not yet been systematically examined. It is also unclear how widespread the filamentous morphology is across influenza virus strains from different host species; filament formation is well established for human IAV and has been observed for porcine strains.^9^, ^15^ Here, we show that the majority of equine IAV strains tested also display a filamentous phenotype in tissue culture and define amino acid residues in M1 that control particle shape.

## Materials and methods

### Cells and viruses

Madin‐Darby canine kidney cells (MDCKs) and human embryonic kidney 293T cells were cultured as described previously.^16^ Equine influenza A viruses were sourced from the World Organization for Animal Health (OIE) reference collection at the Animal Health Trust, Newmarket, UK (Table 1). Human H1N1 A/PR/8/34 (PR8) was either the Cambridge lineage of virus (non‐recombinant) or the MDCK‐cell adapted variant of the NIBSC vaccine strain.^17^ A/Udorn/72 (Udorn: H3N2) was kindly provided by Professor Richard Compans.^11^ The filamentous 7 + 1 reassortant on the PR8 background with segment 7 from Udorn (PR8 MUd) has been previously described.^18^ Passage histories of non‐recombinant viruses, where known, are given in Table 1. Virus stocks were propagated on MDCK cells or in embryonated hens' eggs.

**Table 1:** Equine influenza virus strains used in this study

Virus strain	Abbreviation	Passage history	Accession no.	Comments/reference	Morphology
A/equine/Prague/56 (H7N7)	Prague/56	Unknown	CY130135	Prototype equine H7N7 strain^28^	Filamentous
A/equine/Miami/63 (H3N8)	Miami/63	Unknown	**KF049199**	Prototype equine H3N8 strain^29^	Non‐filamentous
A/equine/Newmarket/79 (H3N8)	Nkt/79	Unknown	**KF049200**	Pre‐divergence equine H3^30^	Filamentous
A/equine/Sussex/89 (H3N8)	Sussex/89	Limited (egg)	**FJ375237**	Pre‐divergence equine H3^31^	Filamentous
A/equine/Newmarket/1/93 (H3N8)	Nkt/1/93	Limited (egg)	**FJ375234**	Prototype American lineage strain^32^	Filamentous
A/equine/Newmarket/2/93 (H3N8)	Nkt/2/93	Limited (egg)	**FJ375235**	Prototype European lineage strain^32^	Filamentous
A/equine/Philippines/2/97 (H3N8)	Phil/97	Limited (egg)	**JN850796**	American lineage	Filamentous
A/equine/Moulton/98 (H3N8)	Moulton/98	Limited (MDCK)	**JN869240**	American lineage	Filamentous
A/equine/Newmarket/11/03 (H3N8)	Nkt/11/03	Limited (egg)	**JN850794**	American lineage^33^	Filamentous
A/equine/Solihull/1/07 (H3N8)	Solihull/07	Limited (egg)	**JN850795**	Florida clade 2 sublineage	Filamentous

‘Limited’ indicates fewer than 5 passages on the indicated substrate; ‘unknown’ passage indicates likely extensive passage potentially on more than one substrate. Accession numbers in bold are for sequences that were generated during the course of this study.

### Microscopy

For immunofluorescence, cells were fixed with 4% formaldehyde in PBS for 20 minute at room temperature and blocked with repeated washes of PBS containing 1% bovine serum. Cell surfaces were stained with rabbit polyclonal sera against whole virus and a fluorescein‐conjugated anti‐rabbit IgG secondary antibody (DAKO), with or without staining of the F‐actin cytoskeleton using tetramethylrhodamine‐labelled phalloidin (Sigma) as previously described.^12^, ^18^ Polyclonal rabbit antiserum was raised against whole A/equine/Sussex/89 (H3N8) virus, while polyclonal rabbit antiserum against whole PR8 virus has been previously described.^19^ Rabbit antiserum reactive against human H3 HA (anti‐X‐31 virus, used to stain Udorn‐infected cells) was the kind gift of Dr David Steinhauer.^12^ Under the conditions employed here (unpermeabilized cells), the antivirion sera primarily stain cell surface HA. Stained cells were visualized using Leica TCS‐NT or Zeiss LSM‐710 confocal microscopes. Projected images of the entire cell surface were generated from stacked images (each Kalman averaged over four repetitions) taken at approximately 0·5 μm intervals through the depth of the cell using Leica tcs software. Post‐capture processing was carried out using Adobe Photoshop. For negative stain transmission electron microscopy (TEM), clarified tissue culture supernatants were applied to glow discharged formvar/carbon‐coated copper grids (TAAB), washed three times in distilled water and stained for 90 seconds in 2% phosphotungstic acid pH 6·8 before drying and imaging on a Phillips CM100 TEM. Scanning electron microscopy (SEM) was carried out as previously described.^12^, ^13^

### RNA extraction and sequencing

Viral RNA was extracted using the QIAamp viral RNA extraction kit according to the manufacturer's instructions. Viral RNA was reverse‐transcribed using 2 μm of segment 7–5′ cRNA (gcgcggtaccGCTCTTCgacccAGCAAAAGCAGGtagatatttaaag) primer and Superscript II RT enzyme and PCR amplified using 10 μm each of segment 7–5′ cRNA and segment 7–5′ vRNA (gcgctctagaGCTCTTCgtattAGTAGAAACAAGGtagttttttactcc) primers and native Pfu polymerase (Stratagene) with 30 cycles of 95°C for 1 minute, 50°C for 1·5 minute and 72°C for 5·5 minute. Sequencing was carried out using BigDye™ Terminator (Applied Biosystems) and gene‐specific primers (available on request) on an ABI Prism 3100 Genetic Analyser (Applied Biosystems).

### Reverse genetics

Segment 7 from equine influenza viruses was amplified using the segment 7 5′ cRNA and 5′ vRNA primers, and the resulting cDNA was cloned into a unidirectional reverse genetics vector pPolI‐SapIRib,^20^ kindly supplied by Professor Ervin Fodor. These plasmids were then transfected into 293T cells along with bidirectional plasmids encoding segments 1–6 and 8 of PR8, kindly supplied by Professor Ron Fouchier.^17^ Point mutations in M1 were created using site‐directed mutagenesis of the relevant segment 7 plasmids using mutagenic oligonucleotides (sequences available on request). The presence of only the desired mutations was confirmed by sequencing of the plasmids and segment 7 from virus stocks.

### Sequence analyses

To generate consensus M1 sequences, the NCBI Influenza Virus Resource (http://www.ncbi.nlm.nih.gov/genomes/FLU/FLU.html) was searched for full‐length M1 polypeptide sequences from avian or equine H3N8 viruses, retrieving 746 avian and 131 unique equine sequences (search performed on 26 July 2013). The multiple alignment tool was then used to generate a consensus sequence.

## Results

**Figure 1:**
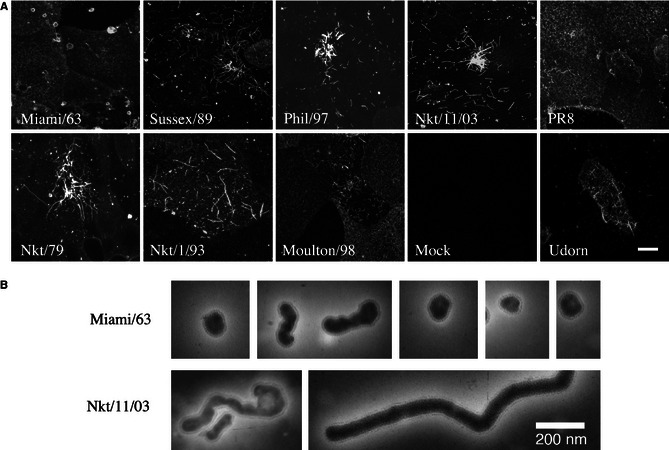
Filament formation by H3N8 equine influenza A viruses. (A) MDCK cells infected (or mock infected) with the indicated viruses were fixed at 16 h p.i., surface stained for viral glycoproteins and examined by confocal microscopy. Images shown are maximum intensity projections of Z‐stacks taken across the depth of the plasma membrane. The mock‐infected cells were stained with the anti‐equine H3 antiserum. Scale bar: 10 μm. (B) Gallery of virus particles visualized by negative stain transmission electron microscopy.

To determine whether equine influenza viruses produce filamentous particles, MDCK cells were infected with a panel of H3N8 virus isolates spanning over 40 years (Table 1) as well as with representative filamentous (Udorn) or non‐filamentous (PR8) human virus isolates. As expected, cells infected with Udorn showed micrometre‐long filamentous structures on their surface that stained brightly for the viral HA (Figure 1A). In contrast, viral antigen was localized much more diffusely on the surface of PR8‐infected cells, and filamentous structures were rarely seen. The HA on cells infected with the prototype H3N8 equine influenza strain, Miami/63, was similarly diffusely distributed with no obvious filaments present. Most other equine viruses examined produced striking filamentous structures that approached 10 μm in length (*e.g*. Nkt/79, Sussex/89 and Nkt/11/03 in Figure 1A), whereas some produced filaments that were around 3–5 μm long at most (*e.g*. Phil/97 and Moulton/98 in Figure 1A) (summarized in Table 1). To test whether the differences in morphology held true for released virus particles, cell‐free material from Miami/63 and Nkt/11/03 was examined by negative stain electron microscopy. Miami/63 virions were a mixture of near‐spherical particles around 100 nm in diameter and more pleomorphic ‘bacilliform’ structures (Figure 1B). No virus particles with a long axis of greater than approximately 250 nm were observed. In contrast, Nkt/11/03 virions were visualized as a mixture of bacilliform particles, highly elongated and irregular particles and clearly filamentous virions of consistent diameter but lengths approaching 1 μm.

**Figure 2:**
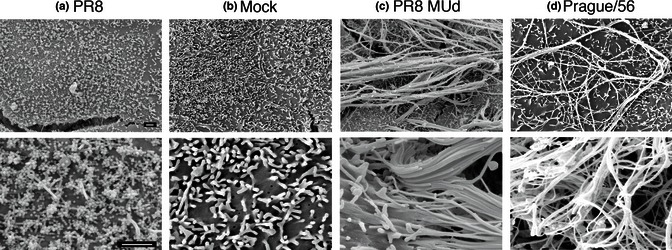
Filament formation by the prototype H7N7 equine influenza A virus. MDCK cells were infected with the indicated viruses at an MOI of 3, fixed at 14 h p.i. and imaged by SEM. Scale bars: 1 μm.

As no suitable anti‐H7 antiserum was available for immunofluorescence, the morphology of Prague/56 was determined by SEM. PR8 and PR8 MUd viruses were used as controls. The surface of PR8‐infected cells was profusely decorated with around 100‐nm‐diameter spherical structures, clearly distinguishable from the generally wider and longer microvilli present on the surface of uninfected cells (Figure 2A,B). In contrast and as previously observed,^13^ the surfaces of PR8 MUd‐infected cells were decorated with bundles of long (in some cases > 20 μm) parallel‐sided viral filaments (panel c). Similar structures, albeit generally less densely arrayed, were observed on Prague/56‐infected cells (panel d), indicating that it too made filamentous virions.

**Figure 3:**
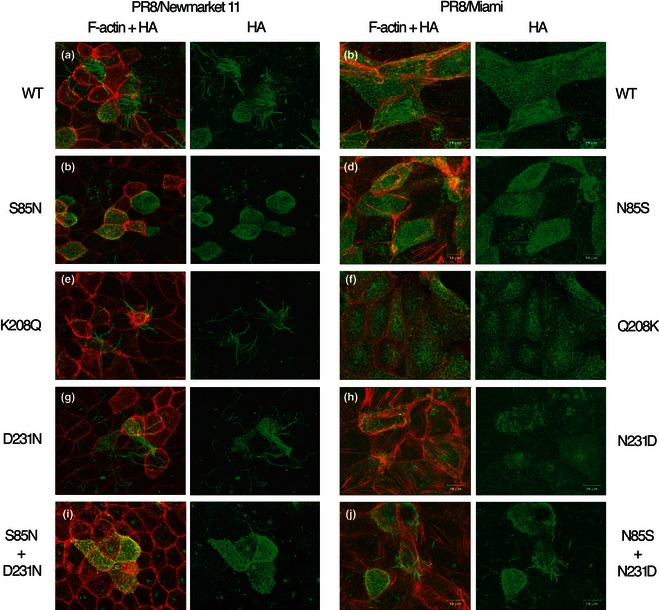
Effect of M1 amino acid polymorphisms on budding morphology. MDCK cells were infected with the indicated viruses at an MOI of < 1. fixed at 16 h p.i and stained for F‐actin with Alexa 594‐phalloidin (red) and cell surface HA (green) before imaging by confocal microscopy. Maximum intensity projections of the cell surface are shown.

To determine the role of segment 7 in equine influenza virus morphology, ‘7 + 1’ reassortants with 7 gene segments from non‐filamentous PR8 and segment 7 of Nkt/11/03, Nkt/1/93, (examples of filamentous) or Miami/63 (non‐filamentous) were generated, and their budding morphology was determined by immunofluorescence. The PR8 + Miami reassortant retained the stippled surface HA staining pattern typical of a non‐filamentous virus, whereas the PR8 + Nkt/11/03 reassortant produced long bundles of filamentous virions (Figure 3A,B). The filamentous phenotype could similarly be transferred to PR8 by segment 7 from the Nkt/1/93 and Sussex/89 strains (data not shown).

**Figure 4:**
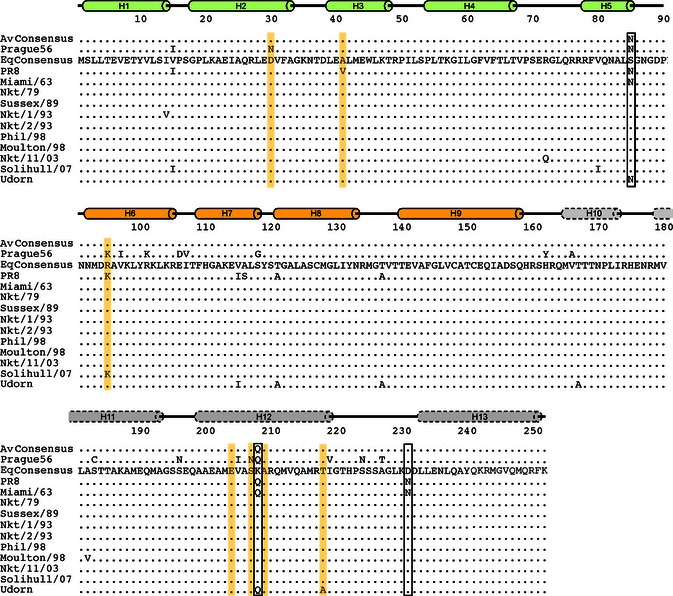
Alignment of equine IAV M1 sequences. Predicted amino acid sequences of the indicated M1 polypeptides are aligned to a consensus sequence of H3N8 equine M1 polypeptides. A consensus sequence for avian H3N8 M1 proteins is also shown. Residues shown to affect budding morphology in human and/or porcine IAV strains are highlighted by yellow shading. Amino acid differences between the non‐filamentous Miami/63 and consensus sequence are boxed. Cylinders above represent known (solid lines) or predicted (dashed lines) α‐helices in M1 from the N‐terminal (green), middle (ochre) and C‐terminal (grey) domains of the protein.

Sequencing of segment 7 of the panel of equine influenza viruses revealed that the predicted amino acid sequence of the non‐filamentous Miami/63 M1 protein differed from the consensus equine virus M1 sequence at only three positions: S85N, K208Q and D231N (Figure 4, boxed). Interestingly, two of the three differences (S85N, K208Q) were conserved between Miami/63 and the consensus sequence for avian H3N8 M1 proteins. Notably, K208Q is in the middle of a cluster of residues within a predicted α‐helix in the C‐terminal domain of M1 found by previous studies to affect virion morphology.^7^, ^8^, ^9^, ^21^ However, all the equine viruses, including the non‐filamentous Miami/63, possessed the ‘signature’ filamentous amino acid sequences identified for human H1N1 and H3N2 strains of virus 7, 8 at positions 41, 204 and 218. Furthermore, most (with the exception of the filamentous Prague/56 and Solihull/07 viruses) also matched the filamentous consensus at position 95 (Figure 4). Similarly, all the H3N8 viruses contained the D30, S207 and A209 residues recently identified as defining filamentous/non‐filamentous morphology in swine‐derived 2009 pandemic viruses.^9^

To determine which of the M1 polymorphisms seen in Miami/63 affected particle shape, a set of single amino acid substitutions was introduced into the PR8 reassortant viruses altering the Nkt/11/03 sequence to the Miami/63 sequence or *vice versa*. Most mutant viruses were readily rescued and grew to high titres (data not shown), but the D231N change on the Nkt/11/03 background was difficult to rescue and grew very poorly, restricting subsequent analyses. When the mutant viruses were tested for their budding phenotype by immunofluorescence, K208Q and D231N substitutions did not have a major effect on the budding of the PR8 + Nkt/11/03 virus, but an S85N change drastically reduced the formation of long filamentous structures (Figure 3C,E,G). Conversely, an N85S change was not sufficient to confer an obvious filamentous phenotype on PR8 + Miami and nor was the Q208K change (Figure 3D,F). The PR8 + Miami N231D virus, however, produced small filamentous structures (Figure 3H). As single mutations had limited effects, the effects of pairs of changes were tested. A combination of N85S and Q208K on the PR8 + Miami background remained non‐filamentous (data not shown), whereas a virus containing N85S and N231D now produced more obvious bundles of filaments, albeit still shorter than with PR8 + Nkt/11/03 (Figure 3J). Thus, in equine segment 7, the identities of M1 amino acids 85 and 231 are crucial determinants of the ability to form large filamentous structures on the cell surface.

**Figure 5:**
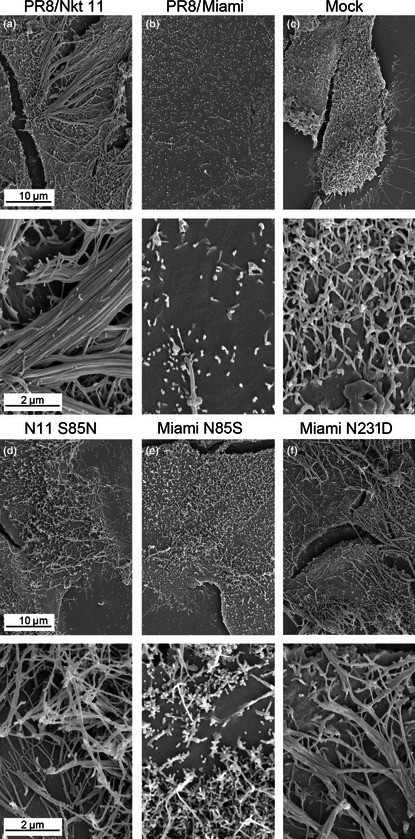
SEM analysis of single M1 polymorphisms of budding phenotype. MDCK cells were infected (or mock infected) with the indicated viruses at an MOI of 3, fixed at 14 h p.i. and imaged by SEM.

Immunofluorescent labelling of the cell surface is a convenient way of assessing the ability of a virus to make large bundles of long filaments, but does not detect shorter filaments. Accordingly, to assess more subtle phenotypic changes, the budding morphology of selected viruses was examined by SEM. Cells infected with the parental PR8 + Nkt/11/03 virus displayed obvious clusters of multiple (often 20 or many more) filamentous virions in parallel array budding from a discrete foci on the plasma membrane (Figure 5A), which plausibly represent the large spike‐like structures seen by immunofluorescence. In contrast, PR8 + Miami‐infected cells were decorated with large numbers of small globular structures approximately 100 nm across with only the occasional small filamentous structure, as expected for a strain that largely produces spherical particles (Figure 5B). In addition, the PR8 + Miami‐infected cells were largely devoid of the microvilli seen on the mock‐infected cells (Figure 5C). By SEM, it could be seen that the introduction of the S85N change into the Nkt/11/03 M1 protein did not totally abolish filament formation, but drastically reduced their number, such that large parallel bundles were largely absent (Figure 5D). Although the reciprocal change (N85S) in PR8 + Miami clearly increased the number and length of filamentous cell surface structures compared with the parental virus, the majority of the budding viruses were still in the form of spherical particles (Figure 5E). However, introduction of the N231D change clearly induced the formation of large numbers of filamentous virions (Figure 5F), to similar levels to the PR8 + Nkt/11/03 S85N virus. Thus, single amino acid polymorphisms at positions 85 and 231 of the equine M1 gene play dominant, but not exclusive roles in controlling viral budding morphology.

## Discussion

Here, we show that the ability to form filamentous virus particles in cell culture is a consistent feature of equine influenza viruses. Filamentous strains included H3N8 viruses isolated prior to the divergence of the H3 HA into European and American lineages, isolates from both the latter groups and from the currently circulating Florida clade 2 sublineage. We also show that for equine influenza, similarly to other species of IAV, segment 7 and in particular, the M1 gene plays a primary role in determining budding morphology. However, the precise genetic determinants of virion shape are not the same between human, porcine and equine strains of virus. By reference to previous data,^7^, ^8^, ^9^, ^10^ the non‐filamentous Miami/63 strain might have been predicted to be filamentous by virtue of the sequence motifs V41, K95, E204, S207 and A209, with only T218 arguing for a non‐filamentous phenotype. Conversely, the filamentous Prague/56 isolate might have been expected to be non‐filamentous, possessing N30, K95, N209 and T218 sequences. Thus, predicting the budding morphology of a specific IAV strain from the sequence of its M1 gene may not be straightforward. The virus species‐specific differences defined here presumably result from the interactions of the highlighted M1 residues with other sequence polymorphisms encoded elsewhere in the virus genome. As the filamentous phenotype was transferable from three equine strains of virus to PR8 by segment 7, it is likely that these altered protein–protein interactions are with other regions of M1 and/or the M2 protein, but further work is required to define this.

The single non‐filamentous equine IAV we identified was the prototype H3N8 isolate, Miami/63. This is perhaps analogous to observations made with human IAV, where the PR8 and A/WSN/33 isolates are non‐filamentous, in contrast to the majority of more recent isolates.^7^, ^8^ When tested in the 1940s, 50s and 60s, PR8 was found to be filamentous, and evidence suggests that the non‐filamentous nature of the currently available passages reflects adaptation to growth in the laboratory.^4^, ^15^, ^22^ However, while we do not know the passage history of our Miami/63 isolate in detail, it is notable that the slightly older Prague/56 virus (with an unknown but presumably similar passage history) retained a filamentous phenotype. There was also no evident correlation between numbers of egg passage or cell culture‐only passage and filamentous virion production for the more recent H3N8 isolates (Table 1).

An alternative explanation for the non‐filamentous nature of Miami/63 may be its avian virus‐like characteristics. Phylogenetic analyses of internal genes of Prague/56, including the matrix genes, suggest that it was long established in *equidae*, forming a unique and ancient lineage.^23^ Equine influenza viruses of the H3N8 subtype, on the other hand, are thought to have been introduced into horses directly from an avian source relatively recently before the prototype strain Miami/63 was isolated.^1^, ^24^ Indeed, the consensus sequences of avian and equine H3N8 M1 genes isolated from 1963 onwards are very similar, showing only two amino acid differences (Figure 4). However, Miami/63 matches the avian consensus rather than the equine at both of these positions, one of which is the crucial morphology‐determining residue 85. Infection in mammals, including horses, is usually via the respiratory route, whereas in aquatic avian species, influenza is an enteric disease.^1^, ^25^ This suggests the hypothesis that filamentous budding may be an adaptation to growth in the respiratory tract, potentially for spread within and/or between hosts. In this respect, it would be interesting to examine the budding morphology of A/equine/Jilin/89 (H3N8). This virus was a direct transmission to horses from ducks 26 that caused an extensive outbreak in China, but failed to become established in the equine host.^27^ The M1 polypeptide of Jilin/89 shares the same substitutions as Miami/63 at positions 85 and 208. Overall, therefore, the sequence polymorphisms we identify here provide a knowledge base for future experiments to probe the importance of filamentous particle formation in IAV pathogenesis.

## Addendum

DE and LM designed and performed the majority of the experimental work. EAB performed and interpreted the scanning electron microscopy. HMW, NS and MT performed sections of the reverse genetics experiments. NB, SM and AR generated and analysed virus sequences. JD, DE and PD conceived and directed the project and drafted the manuscript. All authors read and commented on the final manuscript.

## References

[1] Webster RG , Bean WJ , Gorman OT , Chambers TM , Kawaoka Y . Evolution and ecology of influenza A viruses. Microbiol Rev 1992; 56:152–179. 1579108 10.1128/mr.56.1.152-179.1992 PMC372859

[2] Rossman JS , Lamb RA . Influenza virus assembly and budding. Virology 2011; 411:229–236. 21237476 10.1016/j.virol.2010.12.003 PMC3086653

[3] Chu CM , Dawson IM , Elford WJ . Filamentous forms associated with newly isolated influenza virus. Lancet 1949; 1:602. 18124993 10.1016/s0140-6736(49)91699-2

[4] Mosley VM , Wyckoff RW . Micrography of the virus of influenza. Nature 1946; 157:263. 10.1038/157263a0 21016866

[5] Ada GL , Perry BT , Abbot A . Biological and physical properties of the Ryan strain of filamentous influenza virus. J Gen Microbiol 1958; 19:23–39. 13575751 10.1099/00221287-19-1-23

[6] Rossman JS , Leser GP , Lamb RA . Filamentous influenza virus enters cells via macropinocytosis. J Virol 2012; 86:10950–10960. 22875971 10.1128/JVI.05992-11 PMC3457176

[7] Elleman CJ , Barclay WS . The M1 matrix protein controls the filamentous phenotype of influenza A virus. Virology 2004; 321:144–153. 15033573 10.1016/j.virol.2003.12.009

[8] Bourmakina SV , Garcia‐Sastre A . Reverse genetics studies on the filamentous morphology of influenza A virus. J Gen Virol 2003; 84:517–527. 12604801 10.1099/vir.0.18803-0

[9] Bialas KM , Desmet EA , Takimoto T . Specific residues in the 2009 H1N1 swine‐origin influenza matrix protein influence virion morphology and efficiency of viral spread in vitro. PLoS ONE 2012; 7:e50595. 23209789 10.1371/journal.pone.0050595 PMC3507794

[10] Roberts PC , Lamb RA , Compans RW . The M1 and M2 proteins of influenza A virus are important determinants in filamentous particle formation. Virology 1998; 240:127–137. 9448697 10.1006/viro.1997.8916

[11] Roberts PC , Compans RW . Host cell dependence of viral morphology. Proc Natl Acad Sci USA 1998; 95:5746–5751. 9576955 10.1073/pnas.95.10.5746 PMC20450

[12] Simpson‐Holley M , Ellis D , Fisher D , Elton D , McCauley J , Digard P . A functional link between the actin cytoskeleton and lipid rafts during budding of filamentous influenza virions. Virology 2002; 301:212–225. 12359424 10.1006/viro.2002.1595

[13] Bruce EA , Digard P , Stuart AD . The Rab11 pathway is required for influenza A virus budding and filament formation. J Virol 2010; 84:5848–5859. 20357086 10.1128/JVI.00307-10 PMC2876627

[14] Weinheimer VK , Becher A , Tonnies M *et al* Influenza A viruses target type II pneumocytes in the human lung. J Infect Dis 2012; 206:1685–1694. 22829640 10.1093/infdis/jis455 PMC7107318

[15] Choppin PW . On the emergence of influenza virus filaments from host cells. Virology 1963; 21:278–281. 14070184 10.1016/0042-6822(63)90273-3

[16] Carrasco M , Amorim MJ , Digard P . Lipid raft‐dependent targeting of the influenza A virus nucleoprotein to the apical plasma membrane. Traffic 2004; 5:979–992. 15522099 10.1111/j.1600-0854.2004.00237.x

[17] de Wit E , Spronken MI , Bestebroer TM , Rimmelzwaan GF , Osterhaus AD , Fouchier RA . Efficient generation and growth of influenza virus A/PR/8/34 from eight cDNA fragments. Virus Res 2004; 103:155–161. 15163504 10.1016/j.virusres.2004.02.028

[18] Noton SL , Medcalf E , Fisher D , Mullin AE , Elton D , Digard P . Identification of the domains of the influenza A virus M1 matrix protein required for NP binding, oligomerization and incorporation into virions. J Gen Virol 2007; 88:2280–2290. 17622633 10.1099/vir.0.82809-0 PMC2884976

[19] Amorim MJ , Read EK , Dalton RM , Medcalf L , Digard P . Nuclear export of influenza A virus mRNAs requires ongoing RNA polymerase II activity. Traffic 2007; 8:1–11. 17132145 10.1111/j.1600-0854.2006.00507.x

[20] Subbarao K , Chen H , Swayne D *et al* Evaluation of a genetically modified reassortant H5N1 influenza A virus vaccine candidate generated by plasmid‐based reverse genetics. Virology 2003; 305:192–200. 12504552 10.1006/viro.2002.1742

[21] Shishkov AV , Goldanskii VI , Baratova LA *et al* The in situ spatial arrangement of the influenza A virus matrix protein M1 assessed by tritium bombardment. Proc Natl Acad Sci USA 1999; 96:7827–7830. 10393906 10.1073/pnas.96.14.7827 PMC22146

[22] Burnet FM , Lind PE . Studies on filamentary forms of influenza virus with special reference to the use of dark‐ground‐microscopy. Arch Gesamte Virusforsch 1957; 7:413–428. 13521957 10.1007/BF01241959

[23] Ito T , Gorman OT , Kawaoka Y , Bean WJ , Webster RG . Evolutionary analysis of the influenza A virus M gene with comparison of the M1 and M2 proteins. J Virol 1991; 65:5491–5498. 1895397 10.1128/jvi.65.10.5491-5498.1991 PMC249043

[24] Furuse Y , Suzuki A , Kamigaki T , Oshitani H . Evolution of the M gene of the influenza A virus in different host species: large‐scale sequence analysis. Virol J 2009; 6:67. 19476650 10.1186/1743-422X-6-67 PMC2694789

[25] Tellier R . Aerosol transmission of influenza A virus: a review of new studies. J R Soc Interface 2009; 6:S783–S790. 19773292 10.1098/rsif.2009.0302.focus PMC2843947

[26] Guo Y , Wang M , Kawaoka Y *et al* Characterization of a new avian‐like influenza A virus from horses in China. Virology 1992; 188:245–255. 1314452 10.1016/0042-6822(92)90754-d

[27] Guo Y , Wang M , Zheng GS , Li WK , Kawaoka Y , Webster RG . Seroepidemiological and molecular evidence for the presence of two H3N8 equine influenza viruses in China in 1993‐94. J Gen Virol 1995; 76:2009–2014. 7636481 10.1099/0022-1317-76-8-2009

[28] Sovinova O , Tumova B , Pouska F , Nemec J . Isolation of a virus causing respiratory disease in horses. Acta Virol 1958; 2:52–61. 13533033

[29] Waddell GH , Teigland MB , Sigel MM . A new influenza virus associated with equine respiratory disease. J Am Vet Med Assoc 1963; 143:587–590. 14077956

[30] Burrows R , Denyer M , Goodridge D , Hamilton F . Field and laboratory studies of equine influenza viruses isolated in 1979. Vet Rec 1981; 109:353–356. 6275599 10.1136/vr.109.16.353

[31] Binns MM , Daly JM , Chirnside ED *et al* Genetic and antigenic analysis of an equine influenza H 3 isolate from the 1989 epidemic. Arch Virol 1993; 130:33–43. 8503788 10.1007/BF01318994

[32] Daly JM , Lai AC , Binns MM , Chambers TM , Barrandeguy M , Mumford JA . Antigenic and genetic evolution of equine H3N8 influenza A viruses. J Gen Virol 1996; 77:661–671. 8627254 10.1099/0022-1317-77-4-661

[33] Newton JR , Daly JM , Spencer L , Mumford JA . Description of the outbreak of equine influenza (H3N8) in the United Kingdom in 2003, during which recently vaccinated horses in Newmarket developed respiratory disease. Vet Rec 2006; 158:185–192. 16474051 10.1136/vr.158.6.185

